# 16S-rRNA-Based Metagenomic Profiling of the Bacterial Communities in Traditional Bulgarian Sourdoughs

**DOI:** 10.3390/microorganisms11030803

**Published:** 2023-03-21

**Authors:** Vesselin Baev, Elena Apostolova, Velitchka Gotcheva, Miglena Koprinarova, Maria Papageorgiou, João Miguel Rocha, Galina Yahubyan, Angel Angelov

**Affiliations:** 1 Faculty of Biology, University of Plovdiv, Tzar Assen 24, 4000 Plovdiv, Bulgaria; 2 Department of Biotechnology, University of Food Technologies, 26 Maritza Blvd., 4000 Plovdiv, Bulgaria; 3 Institute of Molecular Biology “Acad. Roumen Tsanev”, Bulgarian Academy of Sciences, Acad. G. Bonchev Str., Bl. 21, 1113 Sofia, Bulgaria; 4 Department of Catering and Nutrition, University of Food Technologies, 26 Maritza Blvd., 4000 Plovdiv, Bulgaria; 5 Department of Food Science and Technology, International Hellenic University, P.O. Box 141, 57400 Thessaloniki, Greece; 6 Universidade Católica Portuguesa, CBQF—Centro de Biotecnologia e Química Fina—Laboratório Associado, Escola Superior de Biotecnologia, Rua Diogo Botelho 1327, 4169-005 Porto, Portugal

**Keywords:** traditional sourdough, lactic acid bacteria (LAB), Bulgaria, metagenomics, bacterial communities

## Abstract

Sourdoughs (SDs) are spontaneously formed microbial ecosystems composed of various species of lactic acid bacteria (LAB) and acid-tolerant yeasts in food matrices of cereal flours mixed with water. To date, more than 90 LAB species have been isolated, significantly impacting the organoleptic characteristics, shelf life, and health properties of bakery products. To learn more about the unique bacterial communities involved in creating regional Bulgarian sourdoughs, we examined the metacommunities of five sourdoughs produced by spontaneous fermentation and maintained by backslopping in bakeries from three geographic locations. The 16S rRNA gene amplicon sequencing showed that the former genus *Lactobacillus* was predominant in the studied sourdoughs (51.0–78.9%). *Weissella* (0.9–42.8%), *Herbaspirillum* (1.6–3.8%), *Serratia* (0.1–11.7%), *Pediococcus* (0.2–7.5%), *Bacteroides* (0.1–1.3%), and *Sphingomonas* (0.1–0.5%) were also found in all 5 samples. Genera *Leuconostoc*, *Enterococcus*, *Bacillus*, and *Asaia* were sample-specific. It is interesting to note that the genus *Weissella* was more abundant in wholegrain samples. The greatest diversity at the species level was found in the former genus Lactobacillus, presented in the sourdough samples with 13 species. The UPGMA cluster analysis clearly demonstrated similarity in species’ relative abundance between samples from the same location. In addition, we can conclude that the presence of two main clusters—one including samples from mountainous places (the cities of Smolyan and Bansko) and the other including samples from the city of Ruse (the banks of the Danube River)—may indicate the impact of climate and geographic location (e.g., terrain, elevation, land use, and nearby water bodies and their streams) on the abundance of microbiome taxa. As the bacterial population is crucial for bread standardization, we expect the local bakery sector to be interested in the relationship between process variables and their effect on bacterial dynamics described in this research study.

## 1. Introduction

The unique geographic and climatic conditions in Bulgaria have contributed to the existence of specific lactic acid bacteria (LAB) strains with a pronounced beneficial effect on human health [1]. These strains are found in various traditional fermented foods, including Bulgarian yogurt, sauerkraut, fermented non-alcoholic drinks, and many other products, including baking sourdoughs. Sourdough (SD) tradition has been preserved for centuries in Bulgaria. However, it was almost lost during the second half of the 20th century due to the limited product diversity in bread production in the centralized political and economic system. However, there is a trend of the revival of sourdough technology due to consumers’ increased interest in sourdough bread and bakery products, which have a unique taste, aroma, and structure; have a significantly longer shelf life; and are perceived as “healthier”. This trend is bringing sourdough back to the focus of researchers and modern product development. Sourdough products are currently prepared in isolated households or small bakeries, still preserving old practices, but scientific knowledge about them is lacking. The key to reviving the sourdough tradition for the current market is to study the natural microbial communities of the traditional products and extend this knowledge towards the development of well-formulated starter cultures [2,3].

Sourdoughs are spontaneously formed microbial ecosystems of different species of lactic acid bacteria and acid-tolerant yeasts in food matrices of cereal flours and water, where salt might also be added, as well as herbal or vegetable extracts. Sourdoughs are characterized by complex dynamics of the mixed microbial association during the fermentation process, which results in products with a wide variation in quality characteristics [4,5,6]. Therefore, knowledge of species diversity in sourdoughs and population dynamics plays an essential role in understanding the complex fermentation process and in the selection of strains for new microbial starter cultures to produce high-quality sourdough-based products at an industrial scale [7,8].

Researchers studying sourdoughs have isolated more than 90 LAB species from around the world [9]. However, it is widely observed that a few (two or three) LAB species are predominant in sourdough, which play a crucial role in directing the mutual relationships and/or matrix-specific adaptations in the sourdough ecosystem [10]. LAB contributes to bread quality with various technological and functional properties. They exert a significant effect on the structural characteristics, nutritional value, and production of many metabolites with an impact on the organoleptic characteristics, shelf life, and health-modulating properties of bakery products [11,12]. Recent studies also point out a bidirectional exchange of microorganisms between starters and bakers [13].

Traditional methods for microbial identification rely on phenotypic characterization by staining and culturing. However, the actual composition of sourdough ecosystems remains unknown, together with complex microbial relationships. Therefore, different modern molecular methods are currently being applied for the identification of LAB and yeasts. LAB identification was performed using a combination of phenotypic and genotypic methods [14,15]. Genotypic identification of LAB may be performed by PCR amplification of random polymorphic DNA markers (RAPD) [16,17], repetitive element sequence-based PCR (repPCR), or sequencing of the 16S rRNA gene [18], which is also used to reveal the genetic relationships among bacteria [19]. Yeasts isolated from sourdoughs were identified at the species level by DNA sequencing and analysis of the ITS-5.8S-ITS2 region [20,21]. However, sourdoughs are the product of complex interactions between different microbial groups, and to fully characterize their composition and process dynamics, it is important to consider the entire microbial community rather than individual species.

In recent years, metagenomic approaches, such as NGS sequencing of multiple amplicons of 16S/ITS/28S rRNA, have enabled researchers to characterize sourdough microbiota in full. This methodology provides the possibility to sequence and annotate millions of sequences, thus identifying hundreds of microorganisms harbored in fermented foods [22,23].

In the present study, we aimed to explore the metacommunities of Bulgarian sourdoughs obtained by spontaneous fermentation and maintained by backslopping in bakeries in three geographical areas to gain knowledge about the composition and specificity of the bacterial communities involved in the production of local sourdoughs.

## 2. Materials and Methods

### 2.1. Sourdough Sample Collection

**Figure 1:**
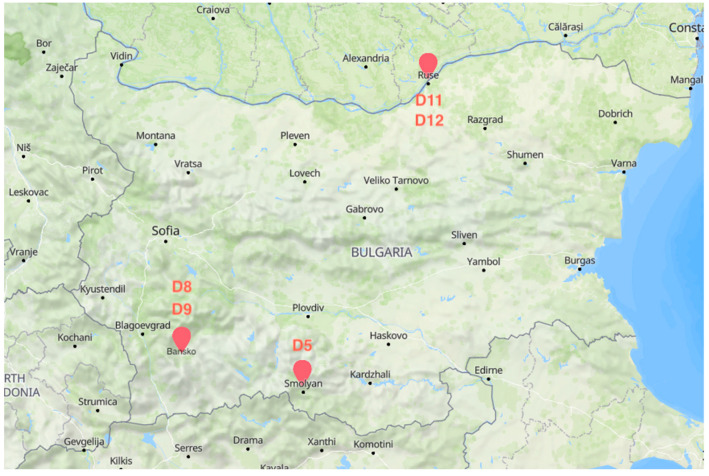
Origin of the sourdough samples—Bulgarian bakery’s locations (D5, Smolyan town, 24.691553, 41.579533; D8, D9, Bansko town, 23.481622, 41.827971; D11, D12, Ruse town, 25.9529, 43.840802).

Samples from five sourdoughs used for the manufacture of typical Bulgarian bread were collected from three bakeries located in different geolocations: Ruse (located in North Bulgaria at the Danube River, at an altitude of 45 m), Smolyan (located in the Rhodope mountains in South Bulgaria, at an altitude of 1002 m), and Bansko (located in Southwest Bulgaria, at the foot of Pirin mountain, at an altitude of 925 m). Information about the geographical origin of the sourdough samples is presented in Figure 1. All samples were taken at the end of the final backslopping, transported, and stored at 4 °C before analysis.

### 2.2. Physicochemical and Biochemical Characterization of The Sourdoughs

All samples were analyzed for dry matter (DM) content, pH, and total titratable acidity (TTA). Dry matter was determined by drying 5 g of each sample at 100–105 °C to constant weight. TTA was analyzed by preparing a suspension of 10 g sourdough in 90 mL distilled water, which was titrated with 0.1 N NaOH to a pH of 8.4, and pH was measured using the pH meter Mettler Toledo FiveEasy FE20. Lactic acid (LA) and acetic acid (AA) content were determined by using the “yellow line” commercial kits of Roche Diagnostics (Basel, Switzerland)—r-Biopharm catalog No. 10 113 9084035 (L-lactic acid in foodstuffs and other materials) and cat. No. 10 148 261 035 (acetic acid in foodstuffs and other materials). All analyses were performed in triplicate and results were expressed with standard deviations. The fermentation quotient (FQ) was determined as the molar ratio between L-lactic and acetic acids [24].

### 2.3. Enumeration of Lactic Acid Bacteria and Yeasts

Enumeration of viable LAB was performed according to ISO 15214:1998 [25]. Each sourdough sample was diluted with peptone water (peptone 10 g/l, NaCl 5 g/l, pH 7.0) and homogenized by Stomacher. Dilutions of 10^−3^, 10^−6^, and 10^−7^ were used for spread-plate inoculation on MRS agar (HiMedia Laboratories Pvt. Ltd., India) for total LAB. The plates were incubated at 37 °C for 72 h under anaerobic conditions (Carbon Dioxide Pack 5–8% CO_2_, Merck KGaA, Darmstadt, Germany). Yeast viable counts were determined by plating serial dilutions on YM-Agar (Oxoid Ltd., Altrincham, UK) and incubating at 27 °C for 48 h. Each sample was analyzed in triplicate.

### 2.4. DNA Extraction

Total DNA was extracted using QIAamp DNA Microbiome Kit (QIAGEN, Hilden, Germany). The protocol involved initial differential lysis and digestion of background (plant) DNA, followed by bacterial cell lysis and isolation of microbial DNA. Extraction was performed according to the manufacturer’s instructions with several optimizations for the specific matrix, the sourdough [26]. DNA quantity and integrity were checked with a Qubit 4 Fluorometer (Thermo Fisher Scientific, Waltham, MA, USA) and agarose gel electrophoresis, respectively.

### 2.5. Sequencing and Data Analysis

The V3–V4 hypervariable region of the 16S rRNA gene was amplified and sequenced using the NovaSeq Illumina platform with a 2 × 250 bp paired-end (PE) read at Novogene (Novogene Europe, Cambridge, UK). The region was amplified using the primer pair 341F (5′-CCTAYGGGRBGCASCAG-3′) and 806R (5′-GGACTACNNGGGTATCTAAT-3′). The amplicon size distribution was qualitatively checked with a 2100 Bioanalyzer (Agilent Technologies, Santa Clara, CA, USA). FastQC software was used to perform a quality check of the sequenced reads and preserve only high-quality sequences (https://www.bioinformatics.babraham.ac.uk/projects/fastqc/, accessed on 30 January 2023). Raw tags were formed by linking the reads from each sample using FLASH (v1.2.71). Chimera reads were identified and discarded using USEARCH [27] as implemented in the QIIME pipeline, and the resulting effective tags were used in the downstream analysis [28]. Operational taxonomic units (OTUs) were picked and clustered using the QIIME pipeline, and taxonomies were assigned based on the SILVA (v132) database at 97% identity cutoff value [29]. OTU abundance information was normalized using a standard of sequence number corresponding to the sample with the least sequences. Downstream alpha (α) and beta (β) diversity analyses were performed based on this output-normalized data. Community alpha diversity indices were calculated using QIIME v1.9.1. UPGMA clustering was performed as a type of hierarchical clustering method to interpret the distance matrix using average linkage and was conducted using QIIME (v1.9.1). A heatmap of the taxonomy was rendered with R package ampvis2 [30].

### 2.6. Data Availability

Raw sequences for each sourdough sample were deposited in GenBank SRA archive and are available with the following accession numbers: BioProject accession number: PRJNA918836; sample accession numbers: SRR23025619, SRR23025618, SRR23025617, SRR23025616, SRR23025615.

### 2.7. Statistical Analyses

All analyses were conducted in triplicate. The obtained data were subject to one-way ANOVA, followed by Tukey’s HSD in R (4.2.1, www.r-project.org, accessed on 30 January 2023). For compact letter display assignment, the multcompLetters4 function was used from package multcompView (0.1). Differences were considered significant at *p*-value < 0.05.

## 3. Results and Discussion

### 3.1. Physicochemical and Microbiological Characterization of Bulgarian Sourdoughs

In the present study, we explored five samples of traditional sourdough collected from three geographical locations: sample D5 from Smolyan town (Rhodope mountains, South Bulgaria), samples D8 and D9 from Bansko town (at the foot of Pirin mountain, Southwest Bulgaria), and samples D11 and D12 from Ruse (Danube Plain, North Bulgaria) (Figure 1). These areas were chosen because they have various geographical and climatic qualities, and the selected local bakeries also have a solid track record of producing traditional Bulgarian sourdough.

All samples were characterized with regards to their dry matter content, pH, total titratable acidity, and lactic acid and acetic acid content. Total viable counts of the two major microbial groups—LAB and yeasts—were also estimated. The results of the sourdough characterizations are presented in Table 1.

The dry matter content ranged from 41.95% (sample D8) to 56.57% (D5), with variations resulting from the raw materials used, sourdough formulation, and manufacturing technology. Dry matter content was positively correlated to the resulting pH of the sourdoughs. The higher water content ensured more free water available and easier flour hydrolysis, therefore a higher amount of fermenting sugars and an easier access of the growing microbiota to the nutrient ingredients, in particular the LAB that produce organic acids. As the dry matter increased, a tendency toward elevated pH was observed: the lowest pH value estimated in sample D8 correlated with the lowest dry matter content, and the highest pH value with the highest dry matter content in sample D5. Vera et al. [31] studied the fermentation dynamics of natural French sourdough, reporting dry matter content of 44.41% at the end of the fermentation, a pH of 3.70, and TTA of 22.4 mL NaOH. While the pH value correlated with dry matter content in a similar pattern as shown in our study, the reported TTA value was much higher than that of the Bulgarian sourdoughs.

According to a recently published review by Calvert et al. [32], sourdough starters should have a pH of 3.9–4.1 and TTA of 14–16 [4]. These reference values vary a little in the literature: a pH of 3.5–4.0 is typically accepted as optimal for sourdough breads [33,34]. Most of our analyzed sourdoughs showed values within this pH range, except for sample D5 (pH 4.28). However, the pH values we observed were similar to the pH intervals of 3.74–4.28 and 3.41–3.70 reported for Italian and French sourdoughs, respectively [4,31].

Regarding TTA, only sample D8 is within the recommended values, with TTA of 15.6. The other four samples had lower TTA values, which could be primarily attributed to the higher dry matter content (that, in turn, influences the dominant microorganisms) but also to other factors such as the number of backsloppings and other parameters employed in the preparation method.

The development of the sourdough fermentation is often evaluated through the fermentation quotient (FQ, i.e., lactic-to-acetic-acid molar ratio). The FQ is a parameter usually used to evaluate the sourness of sourdough bread [4]. In our study, sourdoughs D8 and D9, prepared from wholegrain wheat flour, showed higher FQ values than the samples from white flour—6.31 ± 0.72 and 5.09 ± 0.91, respectively. All samples from white flour had an FQ within the optimal range.

The LAB viable counts of the analyzed sourdoughs were between 9.92 ± 0.96 (D11) and 11.68 ± 0.58 log CFU/g (D5), and the yeast viable counts in the sourdoughs varied from 7.72 ± 0.34 (D11) to 9.59 ± 0.68 log CFU/g (D9). The high TTA could not be linked to the LAB viable counts alone. It may be affected by the homo-/heterofermentative character of the predominant LAB strains in sourdoughs as a major factor for the type and amount of organic acids produced during fermentation, as well as other related endogenous and exogenous baking parameters, such as the fermentation time and temperature, type of cereal and its extraction rate, dough yield, fermentation quotient, number of backsloppings, etc. It was interesting to find that sample D8, which had the lowest DM content, also harbored the highest ratio between LAB and yeast viable counts (2680). This could also be correlated with the lowest pH and highest TTA values recorded for this sample. Samples D5 and D12, which had similar DM values, showed similar LAB-to-yeast ratios, suggesting a link between DM content and the high prevalence of lactic acid bacteria over yeast.

Other studies on sourdough microbiota reported LAB to yeast ratios of 10:1 to 100:1, with 100:1 considered ideal [35,36]. Only one of our studied samples (D9) showed a ratio within this interval—59:1. The other four samples had higher, widely varying ratios, which again could be attributed to the differences in the raw materials and preparation technology.

Organic acids are major metabolites of sourdough fermentation with effects on the gluten network structure and dough elasticity, which also significantly contribute to the specific organoleptic properties of sourdough products compared to other commercially leavened baked products [37,38]. The quantity and type of the organic acids produced during sourdough fermentation depend on various factors such as the microbiota composition and processing parameters (e.g., flour type, dry matter content, dough yield, fermentation time and temperature, and NaCl concentration) [32,39,40].

Many authors have reported higher levels of lactic acid compared to acetic acid in traditional Type-I sourdough starters, which is attributed to the higher number of homofermentative LAB in the sourdough and the lower viable number of acetic acid bacteria (AAB) [41]. The same trend was observed in our study, where LA levels were significantly higher than AA levels in all analyzed samples, from 2.11 times for sample D5, which had the highest DM content (56.57 ± 0.96%), to 6.31 times for sample D8, which had the lowest DM content (41.95 ± 0.47%) (Table 1). It is interesting to note that samples D5 and D12, which had similar DM content, generated the highest AA contents of 37.8 ± 0.9 mM and 29.8 ± 0.8 mM, respectively. These observations also confirm that, generally, the DM of sourdoughs plays a significant role both for the total organic acid production and the ratio between the main organic acids.

The variations in lactic acid and acetic acid in our samples are similar to other reported values of these compounds in sourdough bread [41,42]. The estimated levels of lactic acid in our SD samples varied from 70.9 ± 1.8 to 97.2 ± 1.5 mM (6.38–8.75 g/kg). Debonne et al., (2020) [43] reported a lower LA content of 53 ± 2 mM/kg in Type-C sourdough, while Komatsuzaki et al., (2019) [44] estimated much higher LA values for sourdoughs prepared from wheat and rye flour, reaching approximately 15 mg/g at 8 °C and 30 mg/g at 28 °C. However, in this case, the fermentation was performed with two specially selected natural LAB and yeast strains, which confirmed that the composition of the fermenting microbiota had a major effect on LA formation. Therefore, starter culture selection is essential for the production of sourdough with the desired organic acid levels and profiles.

Acetic acid in our samples ranged from 15.1 ± 1.0 mM (D11) to 37.8 ± 0.9 mM (D5). Debonne et al., (2020) [43] reported similar AA concentrations of 39 ± 1 mM/kg in 1 commercial sourdough, but in 2 other commercial sourdough samples, the AA levels were more than 2 times higher at 89–99 mM/kg sourdough, which was attributed to the presence of *Fructolactobacillus sanfranciscensis*.

Some researchers attribute increased acetic acid levels to yeast activity since yeast releases fructose, which is transformed into acetic acid by heterofermentative LAB [45,46]. Indeed, acetic acid production can be increased by employing obligate heterofermentative LAB and fructose and citrate as alternative electron acceptors [33,34]. However, it was also found that acetic acid inhibits yeast in sourdough more than lactic acid [47,48,49]. In our study, no correlation between yeast viable counts and AA production was observed, which indicates that organic acid production and tolerance are more dependent on LAB, AAB, and yeast species and strains, as reported by other authors [45,50,51]. No correlation between the geographical origin of the samples and their physicochemical characteristics and the estimated LAB and yeast counts was observed. Clearly, the differences found were mainly related to other factors, such as the type of flour used, the recipe, and the preparation method.

### 3.2. Sequencing Data Analysis of Sourdough Samples

The V3–V4 hypervariable region amplicons were sequenced on an Illumina paired-end platform to generate 250 bp paired-end raw reads (raw PE), and then merged (raw tags). The tags were quality-filtered to obtain clean tags. The chimeric sequences in the clean tags were detected and removed to obtain the effective tags used for subsequent analysis.

The sequencing depth of the V3–V4 amplicons we achieved in the 5 sourdough samples was between 158 and 166K raw PE reads. A summary of each data processing step is shown in Supplementary Table S1. The percentage of obtained effective tags for the V3-V4 sequenced region varied in the 5 samples between 73.56 and 81.53%, with a quality score Q30 between 93.58 and 94.13%. The average length of the tags was similar to that of the target region.

To determine the microbial community composition in each sourdough sample, OTUs were obtained by clustering with 97% identity on the effective tags of all samples and were then identified. We observed a large number of tags that were annotated (Taxon tags) and a very low number of unannotated tags in the five samples. The average number of identified OTUs was 816 (between 667 and 1164) (Supplementary Figure S1).

### 3.3. Bacterial Community Diversity within the Sourdough Samples (Alpha Diversity)

The total number of high-quality OTUs obtained per sample was used to calculate alpha diversity indices and rarefaction curves. Rarefaction curves are widely used for indicating the biodiversity of samples and directly reflect the rationality of the sequencing data volume and indirectly reflect the richness of microbial community in the samples (if sequencing depth is enough to capture all the diversity). The rarefaction curve showed that the data of all the samples approach completeness in the curve plateaus, and most of the OTUs were identified (Supplementary Figure S2). The microbial alpha diversity, including the Simpson index, Chao1 index, Shannon index, ACE index, and observed number of species, was estimated and is presented in Supplementary Table S2. The Chao1 and ACE indices were employed to identify the richness of microbial communities, whereas the Shannon and Simpson indices were used to identify the diversity of microbial communities. There was a significant difference in alpha diversity among samples D5 and D8, which showed higher richness values (Chao1 and ACE) and identified numbers of OTUs compared to the other three samples.

### 3.4. Bacterial Communities in the Studied Sourdough Samples

**Figure 2:**
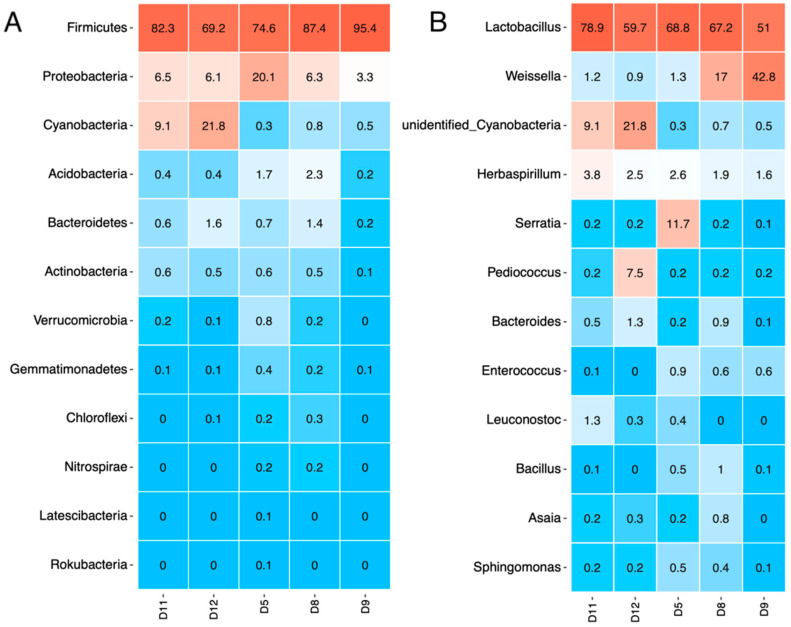
Taxa relative abundance at the phylum level (**A**) and genus level (**B**) in the studied sourdough samples.

The bacterial sequences from 16S rRNA genes assigned to bacterial phyla and their relative abundance (Figure 2A) varied slightly between the samples. As expected, all mature sourdough samples were dominated by Firmicutes phyla (69.2% to 95.4%), with a lower abundance of Proteobacteria (3.24% to 20.05%).

V3-V4 regions have been mostly used for identifying 16S rRNA gene sequences [52,53]. An increased presence of *Cyanobacteria* spp. was found in samples D11 and D12. Since these two sourdoughs originated from the same bakery, Cyanobacteria could have been introduced through the use of contaminated water (water source, pipelines, or containers). Several studies on cyanotoxin contamination of food have found evidence of Cyanobacteria in aquatic products, grains, fresh produce, dietary supplements, and maize [54,55,56], with the usual contamination route of irrigation with contaminated water or the use of contaminated water during food processing. It has been demonstrated that Cyanobacteria contamination can quickly occur throughout the entire food chain [57,58].

Most of the OTUs were classified at the genus level and are shown in Figure 2B. *Lactobacillus* was the main genus found in all sourdough samples (D5—68.77%, D8—67.24%, D9—51.02%, D11—78.92%, and D12—59.73%). The genus *Pediococcus* was present at very low levels (0.2%), with the exception of sample D12 (7.53%). Menezes et al. (2020) [23] also reported a low relative abundance of *Pediococcus pentosaceus* (0.02%) in Brazilian sourdoughs, whereas *Pediococcus* was the dominant genus in western China sourdoughs [59]. Similarly, the genus *Pediococcus* was reported as the most abundant genus in wheat-based sourdoughs in Iran [60].

Interestingly, *Weissella* was the second most predominant genus of LAB found in the wholegrain samples D8 and D9 (from the same bakery in Bansko), with 16.98% and 42.84%, respectively. In comparison, it was found in low abundance (<1.5%) in the rest of the samples made of white flour. In this case, the origin of the same bakery is related to the presence of *Weissella* representatives. At the same time, the two samples differed in the relative amount of *Weissella*, which may be related to the different wheat species (*T*. *aestivum* and *T*. *monococcum*) used for the wholegrain sourdough. *Weissella confusa* has been isolated from various habitats, including the skin, milk, and feces of animals; human saliva, breast milk, and feces; and, in particular, in starchy or cereal-based foods [61,62]. Based on these habitats, its presence in traditional Asian and African fermented foods and in European sourdoughs is quite common, as reported by several authors [10,63,64]. *Weissella spp*. are attracting much interest among researchers since various strains have been found to exert probiotic potential, and others can produce valuable oligosaccharides with prebiotic properties and polysaccharides with biotechnological applications. However, some representatives of this species (*W*. *cibaria* and *W*. *confusa*) have been recognized as opportunistic pathogens [62]. Therefore, identification at the species level of *Weissella* spp. found in samples D8 and D9 would be necessary in order to assess whether the present strains would have a beneficial or detrimental effect.

The genus *Serratia* was found in high abundance (11.73%) in 1 of the samples (D5), while in all other samples the amount was below 1%. Since this sample is from a different location to the other four samples, the presence of *Serratia* sp. may be related to the microflora specific to the producing bakery. *Serratia* spp. are widely distributed in nature and have been isolated from the soil [65]. Therefore, their presence in a hospital environment and different foods—dairy, meat, and fish products [66,67]—is associated with breaches in hygienic practice.

*Herbaspirillum* spp. were found in all the samples, with higher abundance levels of between 2.5% and 3.8% in samples D5, D11, and D12 and lower levels in the wholegrain samples D8 and D9. The presence of *Herbaspirillum* species is closely associated with plants, both endophytically and epiphytically. These bacteria were isolated from different cereals, such as rice, maize, and sorghum, which might indicate a route for their presence in sourdoughs [68]. In addition, studies by Barton et al., (2006) and Salter et al., (2014) [69,70] found that *Herbaspirillum* spp. were among the contaminating species in PCR reagents and DNA extraction kits, which could also be the reason for their detection in sourdoughs.

Other taxonomic units that fell under the top 12 identified genera (Figure 2) with low abundance in all samples were *Bacteroides* (most dominant with 1.12% in D12), *Leuconostoc* (most dominant with 1.12% in D11), *Bacillus* (<1%), and *Sphingomonas* (0.1–0.5%).

The complete metagenomic profile with identified taxa is provided as an interactive KRONA chart, with which the user can explore each sample at each taxonomic level (http://web.uni-plovdiv.bg/vebaev/16s_metagenomics/KRONA/sd.html accessed on 30 January 2023).

**Figure 3:**
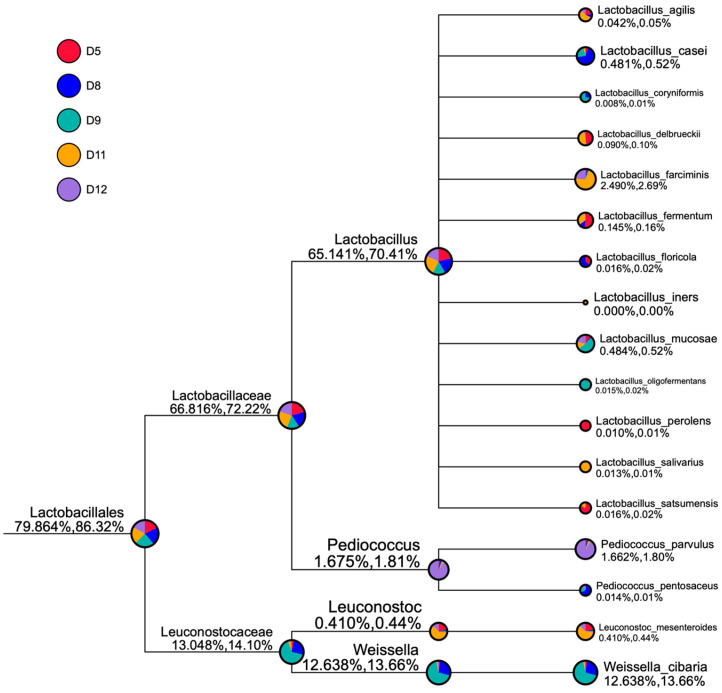
Taxonomy tree profile of the lactic acid bacteria in the studied sourdough samples. The size of the circles represents the relative abundance of species. The first number below the taxonomic name represents the percentage in the whole taxon, while the second number represents the percentage in the selected taxon. Different colors represent different samples. New classification in Lactobacillus taxonomy is as follows: *Ligilactobacillus agilis* (*Lactobacillus agilis*), *Lacticaseibacillus casei* (*Lactobacillus casei*), *Loigolactobacillus coryniformis* (*Lactobacillus coryniformis*), *Companilactobacillus farciminis* (*Lactobacillus farciminis*), *Limosilactobacillus fermentum* (*Lactobacillus fermentum*), *Holzapfelia floricola* (*Lactobacillus floricola*), *Limosilactobacillus mucosae* (*Lactobacillus mucosae*), *Paucilactobacillus oligofermentans* (*Lactobacillus oligofermentans*), *Schleiferilactobacillus perolens* (*Lactobacillus perolens*), *Ligilactobacillus salivarius* (*Lactobacillus salivarius*), *Liquorilactobacillus satsumensis* (*Lactobacillus satsumensis*).

Regarding the profile of LAB, which tend to be the most critical group for sourdough matrices, fewer reads were classified to the species level (Figure 3).

The diversity of LAB in sourdoughs has been found to depend on several factors, such as the raw materials, geographical origin [60,71], preparation technology, microbiota at the bakery (production environment), human microbiota, and methods applied for sampling and identification. Moreover, the results from next-generation sequencing can be affected by the choice and selectivity of the primers used in targeted approaches, sequencing depth, read length, and the algorithms and databases available and utilized for post-sequencing data analysis [72].

The samples in our study were not representative enough to make conclusions regarding all of these factors. Yet, the results from different samples originating from the same bakery show that the production location (environment) is related to the presence of some common species. For example, *Lacticaseibacillus casei* (*Lactobacillus casei*) and *Loigolactobacillus coryniformis* (*Lactobacillus coryniformis*) were mainly presented in samples D8 and D9 taken from one bakery (Bansko town, Southwest Bulgaria), and *Companilactobacillus farciminis* (*Lactobacillus farciminis*) and *Weissella cibaria* were found in both sourdoughs from Ruse town, North Bulgaria (at the Danube River). Sourdough specificity related to location is an important feature that could be used for sourdough metagenomic authentication. However, we observed that samples from different bakeries prepared from white wheat flour shared common species. For example, *Lactobacillus delbrueckii* was in almost equal proportions in samples D5 (Smolyan, South Bulgaria) and D8 (Ruse, North Bulgaria). *Ligilactobacillus agilis* (*Lactobacillus agilis*), *Limosilactobacillus fermentum* (*Lactobacillus fermentum*), and *Leuconostoc mesenteroides* were the most common in these two sourdoughs. However, some specific species were found only in specific samples, independent of the bakery, such as *Pediococcus parvulus* in sample D12, *Schleiferilactobacillus perolens* (*Lactobacillus perolens*) in sample D5, and *Ligilactobacillus salivarius* (*Lactobacillus salivarius*) in sample D11, which could be attributed to other factors.

Other authors [23,73] have found *Companilactobacillus farciminis* in Italian and Brazilian wheat sourdoughs. It was also isolated from Croatian organic flours and applied as a starter culture in sourdough fermentation to reduce bread spoilage [74]. These diverse locations show that this species is related to the raw materials rather than the geographical factor. Another interesting species, *Lig*. *agilis*, was identified in traditional Iranian wheat sourdough [75] and in the fermented soy-based product tempeh, which indicates the ability of this species to ferment raw materials of different plant origin. Along the grain-feed chain, a strain of *Lig*. *agilis* was isolated from pig manure and showed promising probiotic potential [76].

The former genus *Lactobacillus* was predominant in the studied sourdoughs and had the highest diversity with 13 identified species. *Leuconostoc mesenteroides* (D11 and D5, from different bakeries, but both from white flour) and *W*. *cibaria* (D9 and D8 from the same bakery, both from wholegrain flour) were the only identified species of the respective genera. Other researchers [77,78] found that *W*. *cibaria* was related to the fermentation of sourdoughs from alternative grains such as quinoa, amaranth, and sorghum, which indicates a broader distribution of this species during grain fermentation.

*Pediococcus* spp. were mainly represented by *Pediococcus parvulus* (sample D12), and *Pediococcus pentosaceus* was detected at very low levels in samples D8 and D9 from the same bakery. The occurrence of these two species is not very common in sourdoughs, but it has been reported by other authors [79]. In a review by Landis et al., (2021) [72], *P*. *parvulus* was detected in old sourdoughs. In other fermented products, such as wine, *Pediococcus* spp. produce various enzymes that generate desirable aroma compounds [80]. Immerstrand et al., (2010) [81] explored this species’ potential probiotic and culture-protective characteristics, which indicates the potential for other beneficial applications of strains originating from sourdoughs.

### 3.5. Similarity among the Different Sourdough Samples

**Figure 4:**
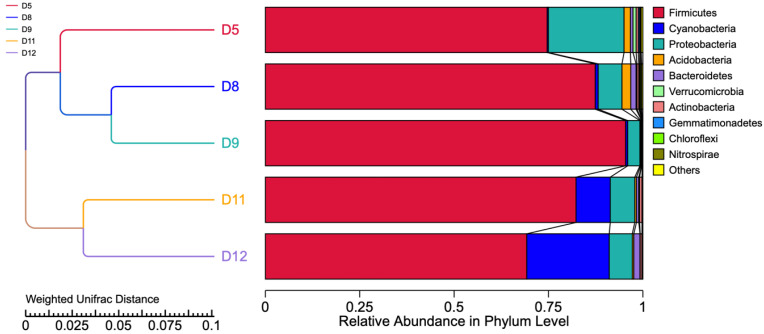
UPGMA cluster tree based on weighted UniFrac distance along with species’ relative abundance and distribution at the phylum level.

A clustering analysis and the construction of a clustering tree were used to investigate the similarities between the different samples. A UPGMA tree based on weighted UniFrac distance at the phylum level is presented in Figure 4. On the left is the cluster tree structure, and on the right is the species’ relative abundance distribution for each sample. These results reveal two main clusters, one composed of D11 and D12 and the other of D5, D8, and D9, with the two latter samples having the greatest similarity.

The UPGMA cluster analysis clearly showed the similarity of species’ relative abundance in the samples from the same location of Bansko town (D8, D9) and Ruse (D11, D12), which shows that the production environment had the most significant impact on the sourdough microbiome in our samples. All five samples were dominated by the phylum Firmicutes, with the highest abundance observed in samples D8 and D9 (bakery in Bansko town, South Bulgaria). Cyanobacteria were found at significant levels in the two samples (D11 and D12) from Ruse town, North Bulgaria. Proteobacteria were also found at similar levels in these two samples, demonstrating the specific effect of location. 

## 4. Conclusions

Sourdough starters are naturally occurring microbial communities in which the environment, ingredients, and bakers are potential sources of microorganisms. Therefore, the characterization of local bakery starters can provide further knowledge, as the relative importance of these microbial pools remains unknown. In this study, we explored five sourdough samples from three Bulgarian bakeries at different geographical locations. *Lactobacillus* was the main genus found in all the sourdough samples. We found that the Pediococcus genus was specific to one of the two samples from the city of Ruse and was present at very low levels in all the others, which did not indicate location specificity. Interestingly, *Weisella* was the second most predominant genus of lactic acid bacteria found specifically in wholegrain samples. Various strains have been found to exert probiotic potential and produce valuable oligosaccharides, but some representatives of this species are recognized as opportunistic pathogens. Therefore, further identification at the species level of *Weissella* spp. found in our wholegrain sourdoughs can highlight these metagenome communities originating from the same geolocation. The genus *Serratia* was also sample-specific, and its presence may be associated with breaches in hygienic practice, which may highlight its importance for product quality and also for human health. 

We further speculate that the presence of two main clusters identified by the UPGMA cluster analysis—one cluster (D5, D8, and D9) including samples from mountain locations (Smolyan and Bansko towns) and the other (D11 and D12) including samples from Ruse town (Danubian river bank)—may indicate the effect of climate and geographical location (e.g., terrain, altitude, land use, and nearby water bodies and their currents) on the microbiome taxonomy abundance.

## Figures and Tables

**Table 1:** Physicochemical, biochemical, and microbiological characteristics of Bulgarian sourdoughs (no baker’s yeast used). Mean values with a different letter in superscript within the same column differ significantly (*p* < 0.05).

Sample Code	Depository	Flour Origin	Dry Matter, %	pH	TTA, ml 0.1 NaOH	LA, mM	AA, mM	FQ	LAB, Log cfu/g	Yeast, Log cfu/g
**D 5**	Bakery “8”, Smolyan (Rhodope mountains, South Bulgaria)	*Triticum aestivum*, *white*	56.7 ± 0.96 ^a^	4.28 ± 0.06 ^a^	12.2 ± 0.12 ^d^	79.7 ± 1.1 ^b^	37.8 ± 0.9 ^a^	2.11 ± 0.82 ^b^	11.68 ± 0.58 ^a^	8.79 ± 0.44 ^ab^
**D 8**	Bakery Samun, Bansko (Southwest Bulgaria)	*Triticum aestivum*, *wholegrain*	41.95 ± 0.47 ^d^	3.56 ± 0.04 ^c^	15.6 ± 0.18 ^a^	97.2 ± 1.5 ^a^	15.4 ± 1.1 ^c^	6.31 ± 0.72 ^a^	11.41 ± 0.88 ^a^	7.99 ± 0.84 ^ab^
**D9**	Bakery Samun, Bansko (Southwest Bulgaria)	*Triticum monococcum*, *wholegrain*	46.67 ± 0.62 ^c^	4.04 ± 0.08 ^b^	13.8 ± 0.16 ^b^	83.5 ± 2.3 ^b^	16.4 ± 0.8 ^c^	5.09 ± 0,91 ^a^	11.36 ± 0.46 ^a^	9.59 ± 0.68 ^a^
**D 11**	Bakery Kusi 1, Ruse (North Bulgaria)	*Triticum aestivum*, *white*	48.78 ± 0.54 ^b^	4.02 ± 0.02 ^b^	13.6 ± 0.08 ^b^	74.5 ± 2.5 ^c^	15.1 ± 1.0 ^c^	4.93 ± 0.88 ^a^	9.92 ± 0.96 ^a^	7.72 ± 0.34 ^b^
**D 12**	Bakery Kusi 2, Ruse (North Bulgaria)	*Triticum aestivum*, *white*	56.35 ± 0.56 ^a^	4.12 ± 0.02 ^b^	12.8 ± 0.12 ^c^	70.9 ± 1.8 ^c^	29.8 ± 0.8 ^b^	2.38 ± 0.84 ^b^	10.81 ± 0.32 ^a^	8.04 ± 0.86 ^ab^

## Data Availability

The data are available from the corresponding author upon reasonable request.

## Supplementary Materials

The following supporting information can be downloaded at: https://www.mdpi.com/article/10.3390/microorganisms11030803/s1, Figure S1. Summary of the annotated tags and OTU numbers of each sample; Figure S2. Rarefaction curves of sourdough samples and identified OTU numbers; Table S1. Statistics of the sample reads raw tags, clean tags, and quality; Table S2. The number of bacterial taxonomic units (OTUs) recovered from the studied sourdough samples and alpha diversity indices.
