# Supplementary material for: 16S-rRNA-Based Metagenomic Profiling of the Bacterial Communities in Traditional Bulgarian Sourdoughs
Source: Microorganisms. 2023 Mar 21;11(3):803. doi: 10.3390/microorganisms11030803 (PMC10058899; doi:10.3390/microorganisms11030803)
Supplement: Supplementary file 1 [file microorganisms-11-00803-s001.zip › Suppl. Figure S1 and S2.pdf]

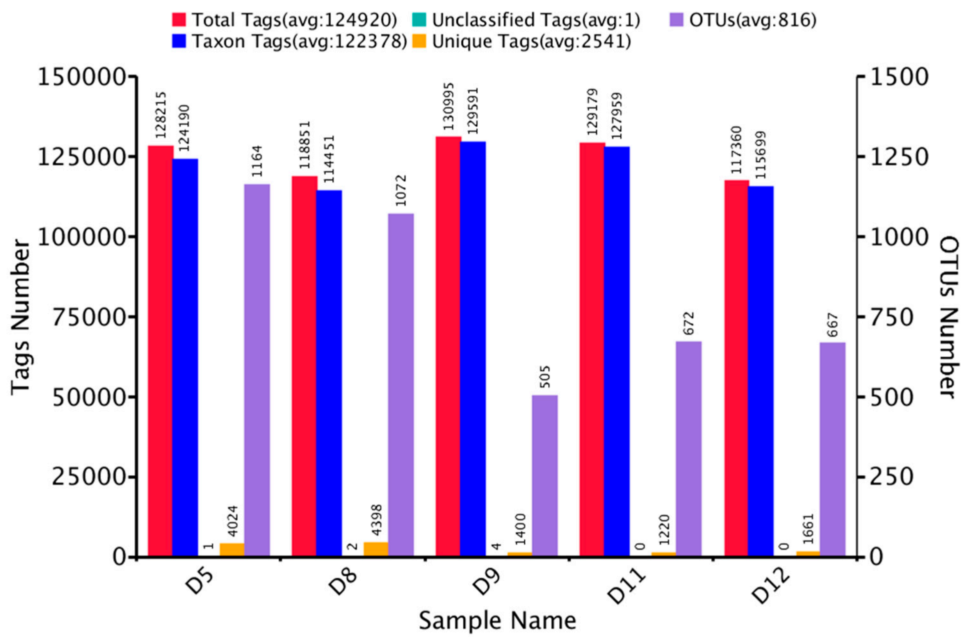

Figure S1. Summary of the annotated tags and OTU numbers of each sample.

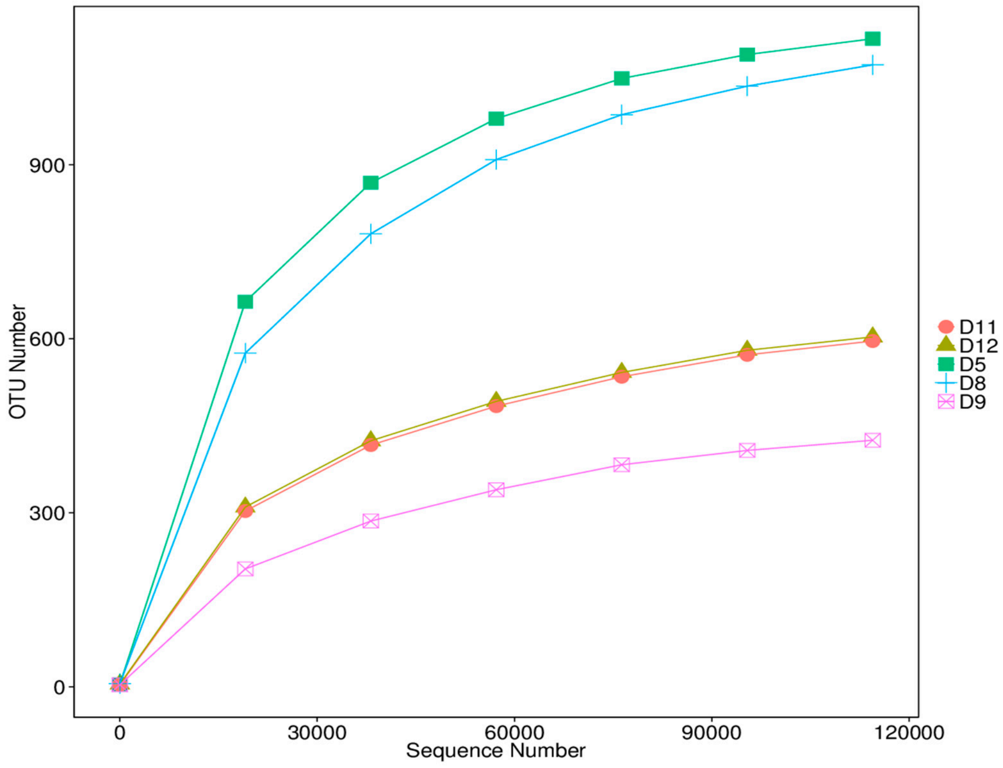

Figure S2. Rarefaction curves of sourdough samples and identified OTU numbers.
