# Supplementary material for: 16S-rRNA-Based Metagenomic Profiling of the Bacterial Communities in Traditional Bulgarian Sourdoughs
Source: Microorganisms. 2023 Mar 21;11(3):803. doi: 10.3390/microorganisms11030803 (PMC10058899; doi:10.3390/microorganisms11030803)
Supplement: Supplementary file 1 [file microorganisms-11-00803-s001.zip › Suppl. Table S1.pdf]

Table S1. Statistics of the sample reads raw tags, clean tags, and quality.

Sample	Raw PE	Raw Tags	Clean Tags	Effective Tags	Avg Len (nt)	Q30
D11	158,838	149,521	147,353	129,179	426	94.13
D12	154,620	138,150	135,848	117,360	423	93.92
D5	165,023	156,804	154,247	128,215	427	93.97
D8	161,563	153,839	151,485	118,851	427	93.94
D9	166,460	157,051	153,854	130,995	429	93.58
