# Supplementary material for: 16S-rRNA-Based Metagenomic Profiling of the Bacterial Communities in Traditional Bulgarian Sourdoughs
Source: Microorganisms. 2023 Mar 21;11(3):803. doi: 10.3390/microorganisms11030803 (PMC10058899; doi:10.3390/microorganisms11030803)
Supplement: Supplementary file 1 [file microorganisms-11-00803-s001.zip › Suppl. Table S2.pdf]

Table S2. The number of bacterial taxonomic units (OTUs) recovered from the studied sourdough samples and alpha diversity indices.

**Sample name**	**OTUs**	**Shannon**	**Simpson**	**Chao1**	**ACE**
D5	1164	3.011	0.579	1157.333	1173.459
D8	1072	3.718	0.824	1145.333	1174.965
D9	505	2.120	0.635	450.744	477.663
D11	672	2.367	0.547	664.303	679.753
D12	667	2.806	0.682	676.714	693.118
